# Early‐life risk factors in Alzheimer's disease and related Dementias

**DOI:** 10.1002/alz70860_105292

**Published:** 2025-12-23

**Authors:** Temitope Kayode, Malika Singh

**Affiliations:** ^1^ New York Medical College, Newark, NJ, USA; ^2^ New York Medical College, Valhalla, NY, USA

## Abstract

**Background:**

Early‐life experiences can shape adult health across a lifetime and potentially across generations, influencing cognitive development and lifelong brain health. The life course approach provides understanding of the impact of childhood experiences on chronic diseases, risk factors and consequences. Evidence suggests that these factors contribute to cognitive reserve, neuroplasticity, and overall resilience to neurodegenerative diseases such as Alzheimer's disease and related dementias (ADRDs). Although there are significant studies on midlife and late‐life risk factors for ADRDs, however, the role of early‐life exposures remains underexplored. The aim of this study is to present evidence of the role of early‐life risk factors in ADRD.

**Method:**

The review synthesizes findings from epidemiologic studies, reviews, retrospective analyses, and neurodevelopmental research that examine the association between early‐life factors and ADRD outcomes. Databases of Google Scholar, Scopus, PubMed were searched to identify relevant studies. Key words used are Early life risk factors, ADRDs, and childhood.

**Result:**

Higher levels of childhood education, intellect, and cognitive stimulation were consistently associated with increased cognitive reserve and a reduced risk of ADRDs later in life (OR0.42; 95% CL,0.37‐0.48). Nutritional deficiencies during critical developmental periods, in iron, iodine, omega‐3 fatty acids, and other micronutrients, were associated with impaired neurodevelopment. Breastfeeding was linked to better cognitive outcomes. ACEs were associated with increased neuroinflammation, hypothalamic‐pituitary‐adrenal (HPA) axis dysregulation, and altered brain structure, such as reduced hippocampal volume, all of which predispose individuals to ADRDs.

Poverty and low parental education significantly impacted brain development and cognitive outcomes, likely through reduced access to educational opportunities and increased exposure to chronic stressors. Neuroimaging studies supported these findings, revealing structural and functional brain changes associated with early‐life adversity, including reduced cortical thickness and impaired white matter integrity.

**Conclusion:**

These findings highlight the significance of early‐life interventions in reducing the long‐term risk of ADRD. Policies at all levels of government should prioritize investments in childhood education, access to adequate nutrition, and mitigating the effects of adverse experiences to strengthen cognitive reserve and reduce susceptibility to neurodegenerative diseases. Addressing socioeconomic disparities during early development could close health inequities gap in ADRD outcomes across populations and the life course

